# Analysis of the Cytoprotective Effect of Amifostine on the Irradiated Inner Ear of Guinea Pigs: An Experimental Study

**DOI:** 10.1016/S1808-8694(15)30520-6

**Published:** 2015-10-18

**Authors:** Ricardo Miranda Lessa, José Antônio Aparecido de Oliveira, Maria Rossato, Thomaz Ghilardi Netto

**Affiliations:** 1 PhD, Assistant Physician - University Hospital - University of São Paulo (USP) Medical School at Ribeirão Preto; 2 Associate Professor. Retired Full Professor - Department of Otorhinolaryngology and Head and Neck Surgery - University of São Paulo (USP) Medical School at Ribeirão Preto; 3 Lab Technician - Department of Otorhinolaryngology and Head and Neck Surgery - University of São Paulo (USP) Medical School at Ribeirão Preto; 4 Associate Professor, Coordinator of the Technical and Administrative Activities of the Radiotherapy Service - University of São Paulo Hospital - Ribeirão Preto. Department of Otorhinolaryngology and Head and Neck Surgery - University of São Paulo (USP) Medical School at Ribeirão Preto

**Keywords:** amifostine, irradiation, inner ear, hearing loss, organ of corti

## Abstract

Radiation can cause damage to the inner ear, from a simple hearing loss all the way to profound deafness. Amifostine is a cytoprotective substance extensively used during radio-chemotherapy for malignant tumors.

**Aim:**

the objective of the present investigation was to establish the antioxidant and radioprotective effects of amifostine on the organ of Corti of albino guinea pigs irradiated in the head and neck region.

**Materials and Methods:**

An experimental study conducted on four groups of guinea pigs were used; One group received only amifostine, one group was submitted to a single dose of 350 cGy and the other two were similarly irradiated but received amifostine doses of 100 or 200 mg/kg. All animals were slaughtered 30 days after the experiment, their bullae were removed and the damaged outer hair cells were counted.

**Result:**

The extent of injury was lower in the outer hair cells of the two groups treated with amifostine compared to the group that was only irradiated. There was no difference between the group treated with 100 and 200 mg/kg of amifostine. The group that received only amifostine had no cochlear damage.

**Conclusion:**

Amifostine is an effective cytoprotective substance in the Organ of Corti of irradiated guinea pigs.

## INTRODUCTION

Radiotherapy is one of the most used treatment modalities against malignant tumors located in the head and neck region. Its mechanism of action on cancer cells is based on de-structuring its nucleotide sequence, thus changing cell molecules, proteins and DNA - affecting cell metabolism and its division mechanism, thus preventing tumor growth.

One of the major drawbacks of head and neck radiotherapy is the very fact that the temporal bone and inner ear structures are within the radiation field of most tumors, as it happens with rhinopharynx tumors. In this case, the hearing organs are inevitably involved in the irradiation field and the patient may develop hearing loss of different degrees, which can even cause profound hearing loss^1^,^2^.

As radiation reaches the vestibulo-cochlear organ, hearing and balance disorders may ensue^3^. Among them, sensorineural hearing loss is the most common complication and it is more commonly associated with the loss of outer hair cells in the organ of Corti^4^.

Many efforts have been made attempting to minimize or neutralize the effects of irradiation to the inner ear; however, the greatest progresses have been seen only regarding the development of new techniques, radiation emission equipment and proton-type power sources, which have reduced the effects of radiation on the hearing organ^5^,^6^.

There are many studies in the literature regarding cytoprotective drugs against the deleterious effects of radiation on normal tissue. Nonetheless, there are almost no studies associated with the action of radiation protection drugs specifically in the inner ear.

One of the major drugs considered for its selective cytoprotective effect and low toxicity against the harmful effects of radiotherapy is amifostine.

The goal of the present investigation was to check whether or not amifostine, administered in the doses of 100 e 200mg/Kg, intraperitoneally, prior to the irradiation dose of 350cGy to the inner ear has any cytoprotective function and, if this protection is significant and selective, prioritizing certain cochlear turns or hair cell groups inside a given cochlear turn.

## MATERIALS AND METHODS

The present study involved 51 male albino guinea pigs weighing between 300g and 450g which passed the positive Preyer reflex hearing screening^7^ test and had distortion products otoacoustic emissions, prior to the study onset, carried out in a System Otodynamics ILO 92 CAE device. The triggering stimulus was of 70dB SPL both for F1 and F2, following the 2F1-F2 frequency relation with F1:F2 ratio equals; 1.22 and resolution of two points per octave.

The animals were broken down into four distinctive groups, which were: Amifostine group - made up of four animals, which received amifostine alone in the dose of 200mg/Kg, intraperitoneally (IP), and served as a negative control for the experiment, since there is no report of ototoxic effect of this drug in the literature; Irradiation Group (Irr) - made up of 11 guinea pigs which received only irradiation to the head and neck at the dose of 350cGy, symmetrically in each ear; Amifostine 100mg+Irradiation (Irr+100mg) Group - made up of 18 animals which received amifostine at the dose of 100mg/kg, IP, 30 minutes before the irradiation of 350cGy; Amifostine 200mg+Irradiation (Irr+200mg) Group - made up of 18 animals which received 200mg/kg, IP, 30 minutes prior to the irradiation of 350cGy. All the groups remained in the experimental surgery lab, complying with the food and housing demands established by the Animal Experimentation Ethics Committee, protocol # 043/2005 approved on March 27, 2006, and the animals were slaughtered 30 days after the experiment.

The method used to compare the amount of damage to the organ of Corti was to count the outer hair cells which were missing in the three rows of cochlear turns: E1, E2, and E3. The parameter used to classify the lesion was the total lack of hairs, in other words, the complete loss of filamentary projection of the hairs on the cuticular plate. The site chosen for cell count was the middle segment of each turn, obtaining the largest possible number of cells within a photographic microscopic field at a magnification of 350 to 700 times. All the turns were photographed and the negatives were developed for later analysis. The numbers resulting from such cell count were turned into fractions of the total number of cells missing, divided by the total population of cells from that photographic field analyzed.

In order to compare the study groups, we held three analyses: the first compared the percentage of damaged cells among the groups (Irr, Irr+100mg e Irr+200mg) considering the percentage of damaged outer hair cells summation on the three turns (E1, E2 and E3) from each group; the second compared the percentage of damaged outer hair cells in each turn (E1, E2 and E3) among the three groups investigated; the third compared the percentage of damaged outer hair cells in each row (F1, F2 and F3) among the three groups.

The results were then submitted to statistical analysis using the Statistical Package for Social Sciences - (SPSS) software. In comparing the values among the three groups we used the Kruskal-Wallis non-parametric test with post hoc test from Dunn when we noticed the existence of significant difference. For this statistical analysis we used p ⩽ 0.05 as level of significance.

## RESULTS

The present paper involved a total of 51 animals, and there were seven deaths. Of these, two happened in the Irr+200mg group, four in the Irr+100mg group, and one in the amifostine group. All the deaths happened because of a diarrhea, very likely of viral origin. From the 44 remaining animals we had 88 cochleas, and at the end of the study we had only 84 -three cochleas were taken off the study because the animals had signs of middle ear inflammation/infection and one cochlea was lost because it was damaged during the dissection.

Since there was no damage in the group which received amifostine alone - which matches literature findings, comparative studies were carried out only among the other study groups.

Comparing the percentage of the total number of OHCs among the groups

**Figure 1:**
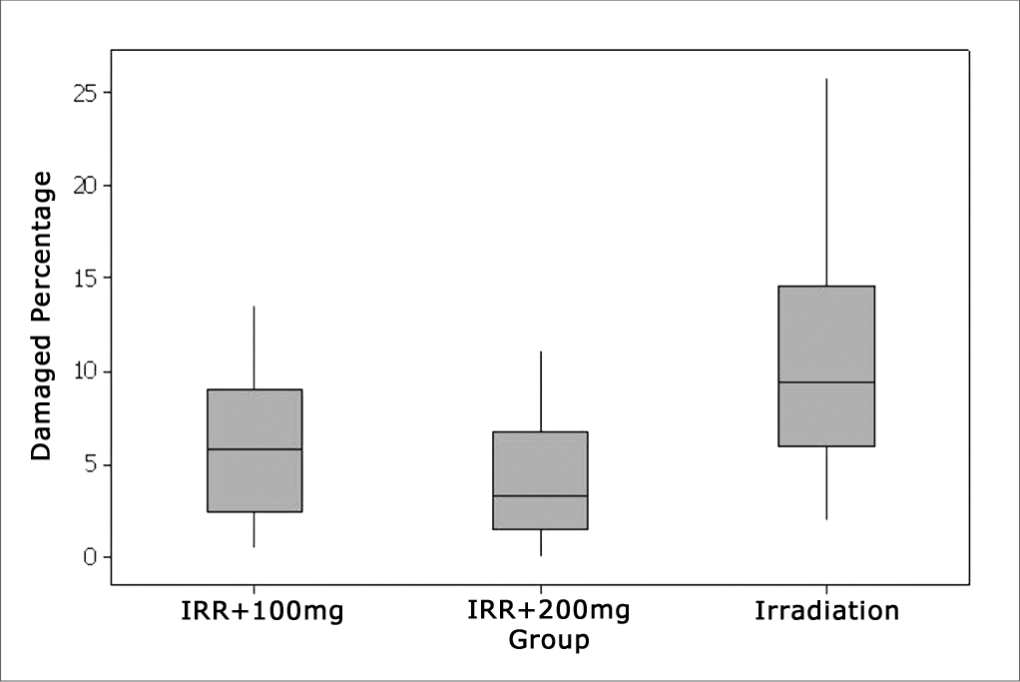
Percentage of damaged OHCs in each group.

Comparing the total number of OHC damaged in each study group it was seen that, in the Irr group the percentage of OHC damage was significantly higher than that in groups Irr+100mg and Irr+200mg, which were equivalent (Figure 1).

Comparing the percentage of damaged outer hair cells in each cochlear turn (E1, E2 and E3) among the groups

### Turn 1

**Figure 2:**
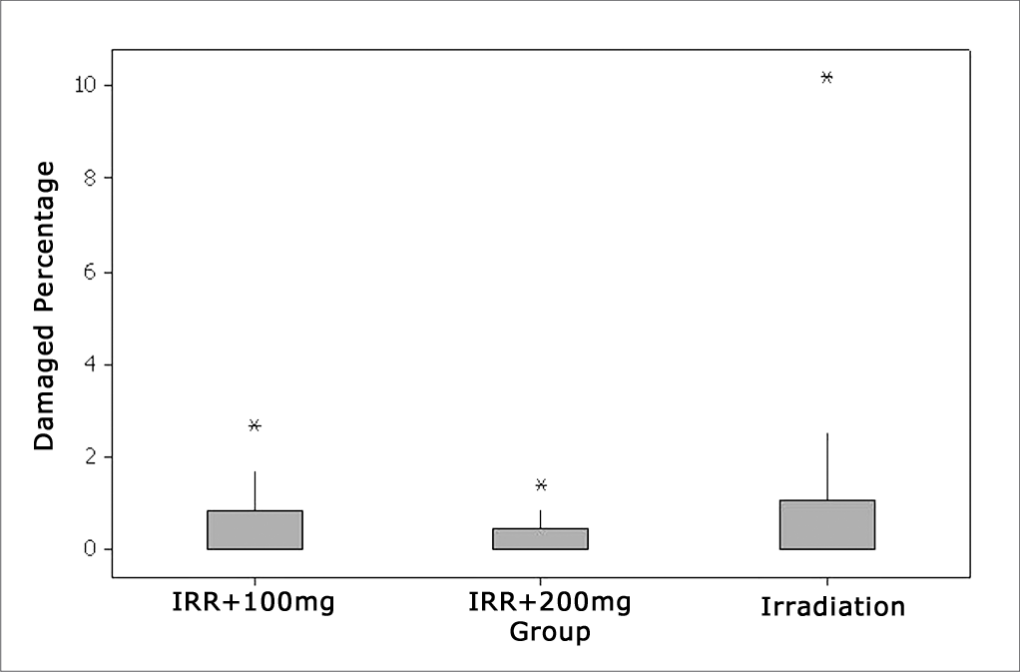
Percentage of damaged OHCs in the first turn.

**Figure 3:**
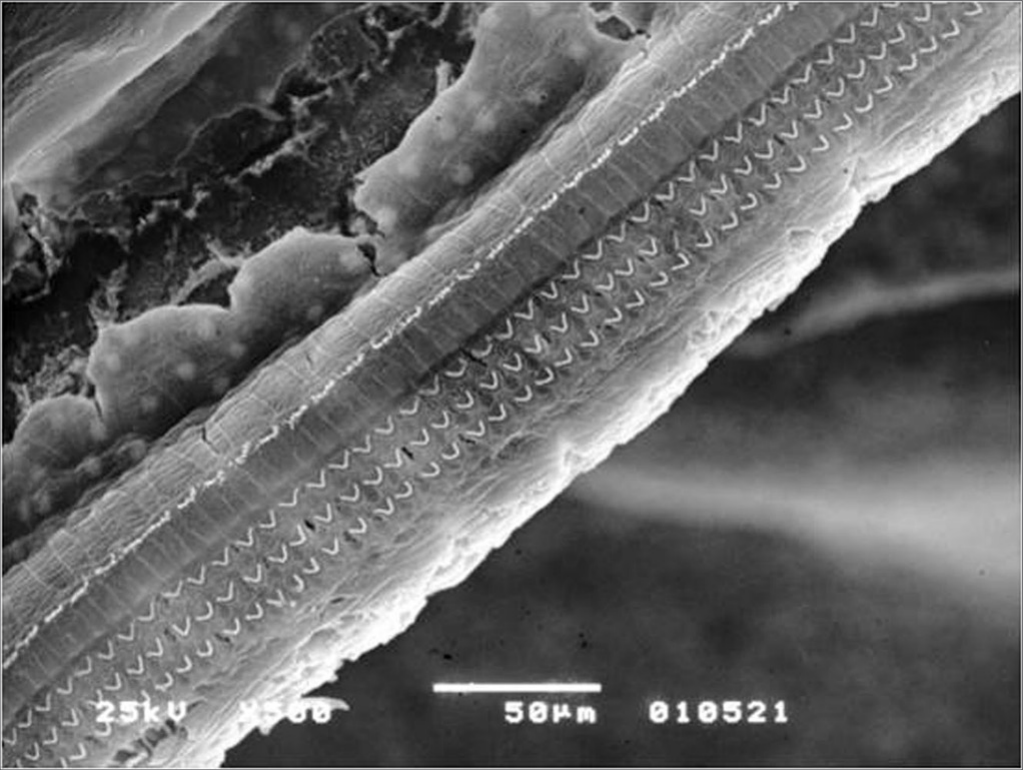
Lesion-free segment of turn 1 in the Irr group.

In cochlear turn 1 we almost did not see any damage in any of the groups studied and, when they did happen, it was in an isolate and inconstant way. Applying the statistic test, there was no significant difference among the groups which received amifostine (100mg/kg and 200mg/kg) before the irradiation and those which were irradiated alone in the dose of 350cGy (Figure 2, Figure 3).

### Turn 2

**Figure 4:**
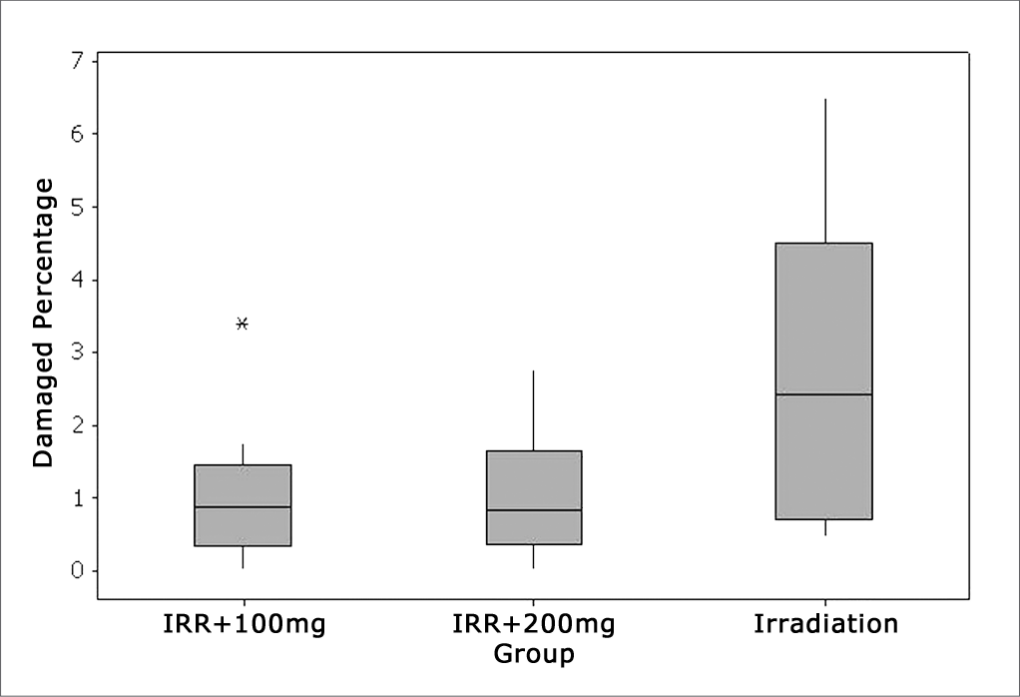
Percentage of damaged OHCs in turn 2.

**Figure 5:**
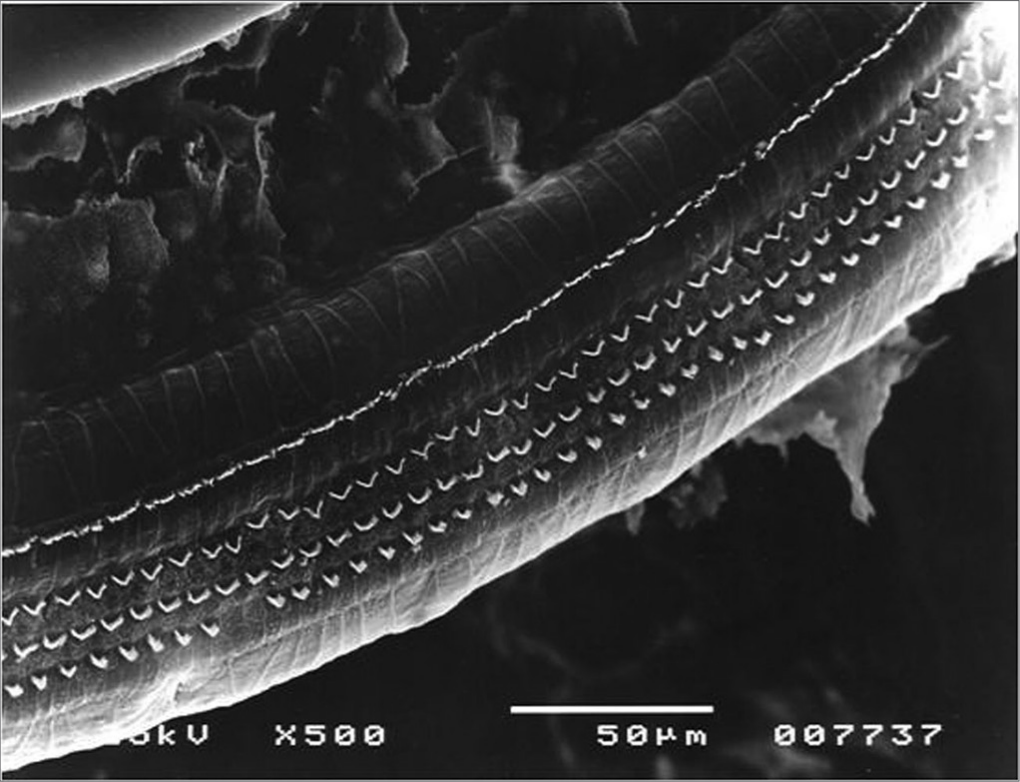
Turn 2 in the Irr+200mg group showing OHC preservation.

In the second turn, we see an increase in blank spots in outer hair cell rows, mainly in the group which did not use amifostine prior to the irradiation. The greater level of damage suffered by this group was statistically significant when the total number of damaged cells in this turn were compared to the other two groups which received amifostine prior to irradiation. Among the groups which received amifostine there was no statistically significant difference (Figure 4, Figure 5).

### Turn 3

**Figure 6:**
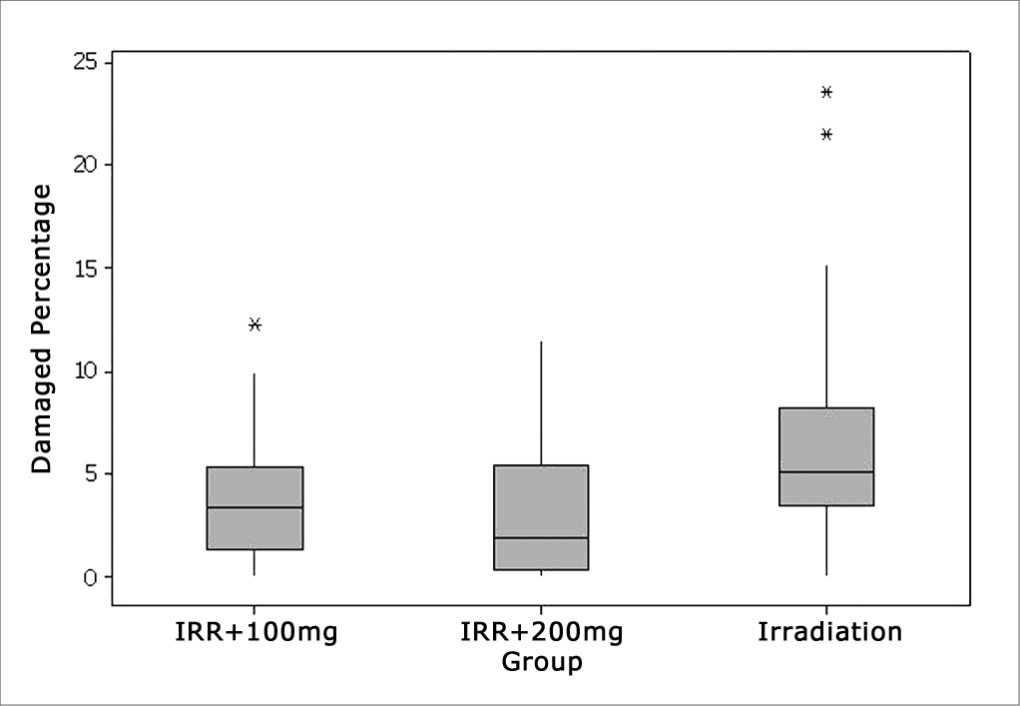
Percentage of damaged OHC in turn 3.

**Figure 7:**
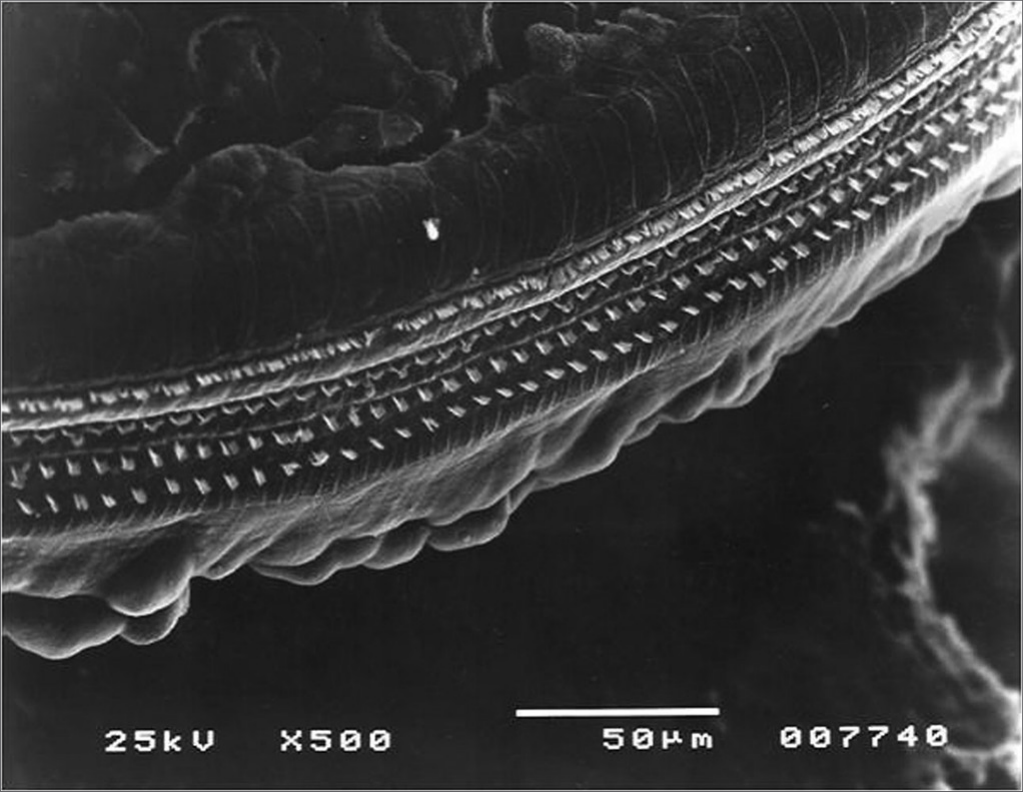
Turn 3 from the Irr+200mg group with OHC preservation.

On the third turn, comparing to E2, there was a higher damage trend in the three groups. Here also, groups Irr+100mg and Irr+200mg showed significantly lower values compared to the irradiated group alone. The two groups which used amifostine were equivalent and did not show significant differences (Figure 6, Figure 7).

Comparing the percentage of damaged outer hair cells in each row (F1, F2 and F3) among the groups.

### Turn 1 - Rows 1, 2 and 3

**Figure 8:**
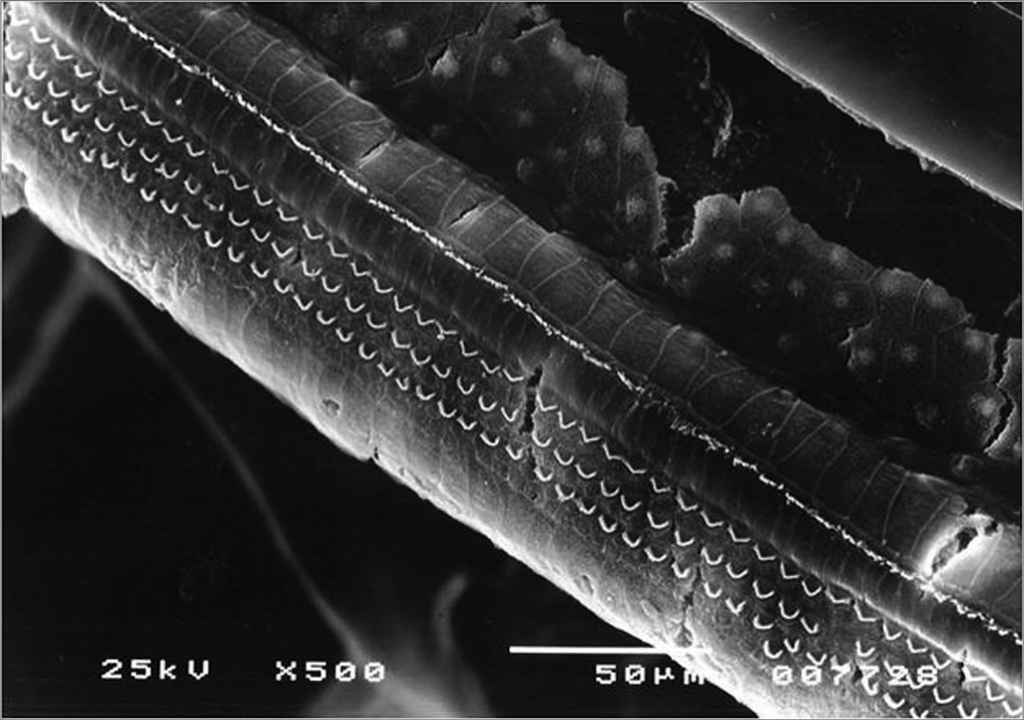
Lesion-free turn 1 of the Irr+200mg group.

In comparing the rows in the first turn, there was no significant difference among the percentages of damaged OHCs in the study groups (Fig. 8).

### Turn 2 - Rows 1, 2 and 3

**Figure 9:**
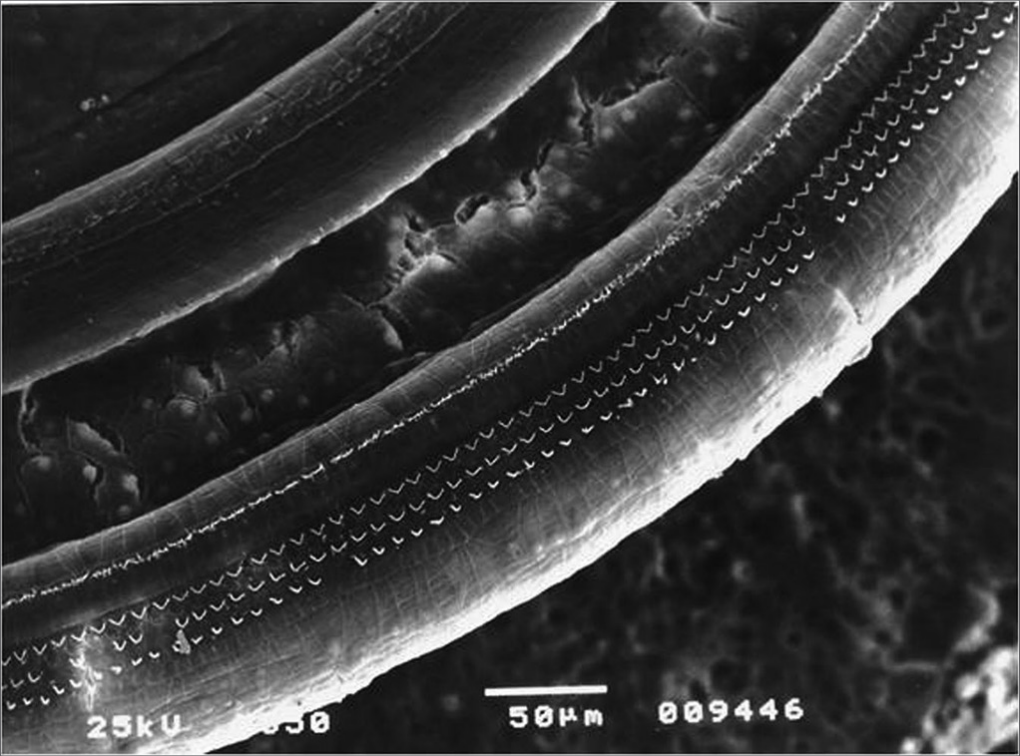
Turn 2 in the Irr group showing F2 and F3 lesion and F1 preservation.

In comparing the percentages of damaged OHCs in the isolated rows in the groups, we observed that, despite the higher damage mean values in the second (F2) and third (F3) rows, it was only in the first row (F1) that we noticed a significant difference by the Dunn post hoc test, in which the group that was only irradiated (Irr) showed significantly higher damage values than groups Irr+ 100mg and Irr+200mg - which were equivalent (Figure 9).

### Turn 3 - Rows 1, 2 and 3

**Figure 10:**
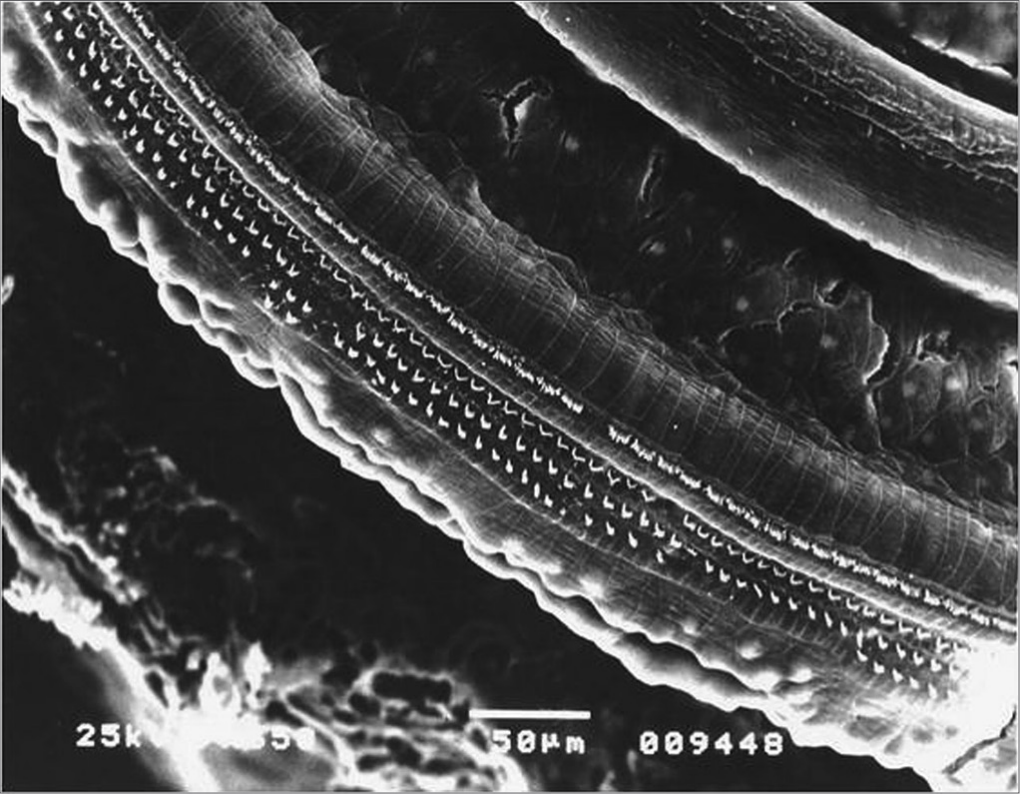
Irr group turn 3 with a large number of hair lesions in F2 and F3.

Comparing the percentages of damaged OHCs among the isolate rows in this turn we noticed a trend towards higher damaged mean values in the second (F2) and third (F3) rows, repeating what happened in row 2. However, only the third row (F3) had significant lesion increase in the group that was only irradiated, compared to the other two groups which received amifostine in the doses of 100mg and 200mg/kg, which were equivalent (Fig. 10).

## DISCUSSION

Radiotherapy-induced hearing loss, despite a very relevant topic, is still not diagnosed enough today and, in a certain way, it is not enough appreciated and prevented among professionals involved with this treatment mode. This is partially due to the lack of knowledge regarding the progress of new radiotherapy techniques with greater ear protection potential and the recent discovery of drugs with radioprotective potential for normal tissue adjacent to the tumor, including the hearing organs.

As to the types of hearing loss induced by radiotherapy, it is known that they can be air-conduction-related, sensorineural and mixed. Sensorineural hearing loss is, without doubts, the most frequent and, according to some authors, it can happen in up to 75% of the patients irradiated in the temporal bone region and it usually develops through the lesion to the organ of Corti and less frequently through an 8th cranial never degeneration, given that central nervous system structures have a greater resistance towards this treatment mode^8^.

As to the dose which is damaging to the organ of Corti, the higher the irradiation dose to the inner ear, the greater is the likelihood of an auditory damage^9^. In general, the total radiation dose necessary to damage the auditory system varies between 3,000 and 6,000 cGy, when divided in small fractions^10^, ^11^, ^12^. In our study, the dose necessary to cause immediate damage to the hair cells was 350cGy, applied in a single dose. Such dose was established based on a pilot study previously carried out in our Department, given that there was no information in the literature about the average damaging dose to the organ of Corti of albino guinea pigs.

Post-radiation hearing loss can be immediate or late^2^. The immediate complication results from the direct cell injury caused by the radiation on the structures of the organ of Corti and the stria vascularis. Now, the late effects caused by the indirect action of radiation on the inner ear vessels, generating obliterating endarteritis process, which progressively interrupts blood supply to vital structures of the cochlea neuroepithelium and the 8th cranial nerve^13^, ^14^, ^15^.

With reference to the frequency range most affected, the low and middle frequencies are rarely affected. The high tones are the ones most affected in the frequency range between 4,000 and 8,000Hz^4^,^15^. Our findings point to the lesion happening preferably on the second and third turns, preserving the first turn and the apical turn. Comparing the lesion degree between E2 and E3 we noticed that the lesion percentage was greater in the third turn, in most of the cases overcoming in two fold what had happened on the second turn. This selective mechanism for the turns and consequently for the hearing frequencies is very likely due to a greater oxygen consumption in the hair cells and stria vascularis closer to the cochlear base, which makes them more sensitive^16^.

Another data also observed was that there was a trend to an increase in the percentage of damaged cells on the second and third turns, especially on the two outermost rows, with preservation of the first and innermost. Such fact also noticed in other experimental studies involving radiation is very likely due to different metabolic needs which happens among these cell groups, already pointed out by some authors who detected differences both anatomical and of biochemical constitution in the outer hair cells in different cochlear sectors^4^,^17^, ^18^, ^19^.

As to the use of ear protection drugs in radiotherapy, Atlas et al. (2006) were able to reduce radiation-induced cochlear damage to the spiral ganglion, stria vascularis, inner and outer hair cells in guinea pigs which were treated with piracetam one hour after irradiation of a 60Gy dose, by the mechanism of cell apoptosis inhibition^20^.

Amifostine is a pro-drug, which is converted by phosphatase alkaline in the WR-1065 active molecule. WR-1065 interacts with free radicals donating hydrogen ions which bind to oxygen molecules and active metabolites of the antineoplastic agents^21^,^22^. This has given Amifostine the status of an efficient cytoprotective in the neurotoxicity, nephrotoxicity, blood toxicity and ototoxicity induced by cyclophosphamide, carboplatin and cysplatin^23^, ^24^, ^25^. Glover et al. (1984) studied the effect of amifostine in the inner ear of patients who received chemotherapy by cisplatin and noticed a reduction in the levels of hearing loss in patients receiving cisplatin in the dose of 150mg/m^2^ ^26^. Hyppolito et al. (2005) compared the number of damaged outer hair cells and the behavior of DPOAE in guinea pigs treated with and without amifostine and the ones which received cisplatin. They concluded that the loss of outer hair cells was lower in the amifostine group and the otoacoustic emissions were present in all the animals of this group after using the ototoxic agent^27^.

In radiotherapy, amifostine protects many cell lineages from the effects of radiation. This drug has already been broadly tested and proved to be effective in the radioprotection of the bone marrow, reducing rates of blood toxicity - minimizing thrombocytopenia, anemia and leucopenia^28^,^29^. In head and neck tumors, it is believed that amifostine can reduce the radiotoxicity to salivary glands, esophageal mucosa and the oral cavity, minimizing the adverse effects of mucositis and xerostomia which substantially compromise the quality of life of post-irradiated patients^30^,^31^.

In our study it was clear that amifostine significantly protected the Irr+100mg and Irr+200mg groups which received the drug intraperitoneally, 30 minutes before irradiating the inner ear, however there was no protection significant difference only by changing the dose from 100mg/kg to 200mg/kg. Such protection was visibly marked in the rate of damaged hair cells and represented a reduction of up to 40% of the lesion percentage of OHCs in the groups that received amifostine. This reduction was probably due to the lesion differences which happened on the second and third turns which, in comparing the groups pointed to a percentage reduction of lesions in the Irr+100mg and Irr+200mg groups, when compared to the Irr groups. In comparing lesion percentage values in turn 1 between the groups, there was no significant lesion difference in any of the groups. This can be explained by the low level of lesion which happened by the radiation in this cochlear sector, in particular.

When the groups were compared in terms of rows, only the first row of E2 and the third row of E3 showed lover and statistically significant values of hair cell lesions in the groups Irr+100mg and Irr+200mg. This is, without doubt a true finding of this study, which must wait for new experiments in order to check whether or not it was a casual phenomenon or something specific for the drug used.

## CONCLUSIONS

Amifostine used prior to inner ear irradiation significantly protected the organ of Corti outer hair cells of guinea pigs irradiated in the regions of the head and neck by Cobalt 60, at the dose of 350cGy and it significantly protected the second and third cochlear turns, and such fact did not happen in the first turn. Only the first row of the second turn and the third row of the third turn showed significantly lower values of hair cell lesion in the groups which received cytoprotection and there was no significant difference in the protective response of the outer hair cells with increase in the amifostine dose from 100mg to 200mg/kg.
